# Risk of Dental Caries and Erosive Tooth Wear in 117 Children and Adolescents' Anorexia Nervosa Population—A Case-Control Study

**DOI:** 10.3389/fpsyt.2022.874263

**Published:** 2022-05-10

**Authors:** Elzbieta Paszynska, Amadeusz Hernik, Agnieszka Slopien, Magdalena Roszak, Katarzyna Jowik, Monika Dmitrzak-Weglarz, Marta Tyszkiewicz-Nwafor

**Affiliations:** ^1^Department of Integrated Dentistry, Poznan University of Medical Sciences, Poznań, Poland; ^2^Department of Child and Adolescent Psychiatry, Poznan University of Medical Sciences, Poznań, Poland; ^3^Department of Computer Sciences and Statistics, Poznan University of Medical Sciences, Poznań, Poland; ^4^Department of Psychiatric Genetics, Department of Psychiatry, Poznan University of Medical Sciences, Poznań, Poland

**Keywords:** anorexia nervosa, adolescence, oral health, caries, erosion, dental plaque, gingival inflammation

## Abstract

**Introduction:**

Restrictive type of *anorexia nervosa* (AN) is still one of the most severe eating disorders worldwide with an uncertain prognosis. Patients affected by AN should be encouraged to undertake psychiatric care and psychotherapy, but whether they should necessarily be included in careful dental care or not may still be questionable. Even though there is a constantly increasing number of AN studies, there are just a few data about the youngest group of AN children and adolescents aged < 18.

**Methodology:**

This case-control study aimed to compare the dental health and gingival inflammation level in female adolescent inpatients affected by severe AN restrictive subtype vs. controls. Based on clinically confirmed 117 AN cases (hospitalized in years 2016–2020 in public Psychiatric Unit, BMI < 15 kg/m^2^, mean age 14.9 ± 1.8), the dental status has been examined regarding the occurrence of caries lesions using *Decay Missing Filling Teeth* (DMFT), erosive wear as *Basic Erosive Wear Examination* (BEWE), gingival condition as *Bleeding on Probing* (BOP) and plaque deposition as *Plaque Control Record* (PCR). The results were compared with age-matched 103 female dental patients (BMI 19.8 ± 2.3 kg/m^2^, age 15.0 ± 1.8, *p* = 0.746) treated in a public University dental clinic.

**Results:**

AN patients were found to present a higher incidence of oral-related complications according to dental status (DMFT 3.8 ± 4.5 vs. 1.9 ± 2.1, *p* = 0.005), erosive tooth wear (BEWE 18.9 vs. 2.9%, *p* < 0.001), less efficient in controlling plaque (PCR 43.8 vs. 13.7%, *p* < 0.001) and gingival inflammation (BOP 20.0 vs. 3.9%, *p* < 0.001) compared with female adolescents. In the AN group, a significant correlation between BOP, BEWE, and duration of AN disease (*p* < 0.05), similarly to the number of decayed teeth D, filled teeth F and PCR were detected (*p* < 0.05).

**Conclusions:**

Although the obtained results did not reveal any severe oral status, our findings indicated impaired dental and gingival conditions in young anorexics. Considering AN's potential role in oral health, it is essential to monitor dental treatment needs and oral hygiene levels in their present status to prevent forward complications in the future.

## Introduction

Restrictive type of *anorexia nervosa* (AN) is still one of the most severe eating diseases worldwide with an uncertain prognosis as 1.2–2.2% of girls/women are suffering from full-blown anorexia nervosa (AN) during their lifetime; even more, than 4% are considered as an atypical form (1–3). Unfortunately, some of the cases are not included in statistics due to a lack of insight into the disease or reluctance to start therapy (2). Anorexic type of eating disorder (ED) often begins quite early, even at 12-years-old or younger (1). A reason why oral status is vital at this age may be explained by their developmental period when the permanent teeth mineralization and periodontal tissue are formulated (4). Any case of oral imbalance may provide long-lasting consequences in their future oral health. Severely ill adolescents with AN are at risk of extremely low body weight, macro/micronutrient deficiencies (5), and combined with diminished salivation and neglecting of hygiene habits, protection for dental or periodontal tissues may be lost at an older age (6–8). Significant dental caries, erosive tooth wear, and loss of periodontal health were observed in other studies considering adult AN subjects (8, 9). However, in the available literature, few scientific reports focused on the oral status among AN individuals under the age of 18 and affected by disease < 5 years, i.e., during the first acute stage of ED (10, 11). It is proven that AN patients should be encouraged to undertake psychiatric/psychotherapy (11). However, whether they should be included in careful dental care may still be considered in an open discussion. Therefore, each oral health assessment among young AN patients may provide important directions for dental care and answer questions about what type of oral care elements should be considered in adolescent AN patients.

The study aimed to establish the oral status regarding caries incidence, tooth wear, gingival inflammation, and oral hygiene levels among severely ill adolescent inpatients diagnosed with AN.

The null hypothesis assumed no significant difference in the oral disease symptoms between patients with AN and healthy individuals matched in early adolescence.

## Methods

### Participants

The case-control study was conducted according to the Good Clinical Practice guidelines and the pattern of the Declaration of Helsinki after approval by the Bioethics Committee of the Poznan University of Medical Sciences (Resolution No. 66/12). The content of the study was explained to all 220 participants, who gave their informed written consent to participate in it. Additionally, a parent or legal guardian's approval was needed for inclusion in the project. A lack of acceptance from patients, parents, or legal guardians excluded such subjects from the study. The subjects were assigned to two groups: the anorexic (AN) group and the control (Ctrl) group (Table 1).

**Table 1:** Inclusion and exclusion criteria for both groups.

**Criteria for inclusion into the study group (AN)**	**Criteria for inclusion into the control group (Ctrl)**	**Criteria for exclusion from study and control groups**
Children of female sex aged 12–18	Children of female sex aged 12–18	Adolescents aged > 18 Male sex
Children with diagnosed AN restrictive subtype in accordance with ICD-10 and DSM-V diagnostic criteria (diagnosis confirmed by two independent psychiatrists)	Lack of mental disorders—assessment with the use of ICD-10 and DSM-V diagnostic criteria (confirmed by two independent psychiatrists). Children without hereditary mental disorders (first-degree relatives)	Children with disorders of the central nervous system (e.g., epilepsy, serious injuries, and CNS infections) Coexisting: schizophrenia, bipolar affective disorder, any serious somatic disorders
Clinically significant AN symptoms lasting over six months	No ED symptoms in present and past times	Chronic somatic diseases Persistent pharmacotherapy Hormonotherapy Contraception Pregnancy Dietary supplements
BMI < 15 kg/m^2^	BMI 17–24 kg/m^2^	BMI > 25 kg/m^2^
A patient, parent or legal guardian approval	A patient, parent or legal guardian approval	Lack of acceptance from patients, parents or legal guardians
N smokers	No smokers	Smoking
	No urgent dental recall visit	Professional scaling Orthodontic treatment Antibiotic therapy Anti-inflammatory drugs 3 months before dental examination

*AN, anorexia nervosa; ED, eating disorders; ICD-10, International Statistical Classification of Diseases and Related Health Problems (10th edition); DSM-V, Diagnostic and statistical manual of mental disorders (5th ed.); CNS, Central Nervous System; BMI, Body Mass Index*.

The AN group consisted of one-hundred-seventeen consecutive adolescent patients from the mid-west part of the country admitted in the acute phase of AN to one public Psychiatric Unit for Child and Adolescents from 2015 to 2020. Patients admitted to the hospital were primarily female because, during the survey, only one boy was hospitalized and suspected to be affected with AN. Still, he was excluded from the study group due to statistical reasons. Diagnosis of the restrictive subtype of AN was confirmed after a semistructured interview conducted by a child and adolescent psychiatrist based on ICD-10 (code F50.1) and DSM-5 (code 307.1) criteria (12, 13). The study group had similar clinical characteristics (restrictive type) concerning their menstrual status (secondary amenorrhea). Other inclusion criteria assumed that the symptoms of the AN disease lasted < 12 months, and only patients who suffered from the main type of AN were enrolled. All patients were hospitalized in the same public Psychiatric Unit for Children and Adolescents for the first time. The medical examination was taken during the acute stage of the symptoms (BMI < 15 kg/m^2^) within the patients' 1st week after admission. The participants did not suffer from any other somatic disorder. A bulimic type of anorexia was excluded from the study to achieve homogeneity among the participants. Other exclusion criteria were: chronic somatic diseases or other mental/neurodevelopmental disorders (a primary disease in relation to eating disorders), hereditary disorders (first-degree relatives), pharmacotherapy, hormonotherapy, pregnancy, contraception, dietary supplements, smoking. Inclusion and exclusion criteria are described particularly in Table 1.

**Figure 1:**
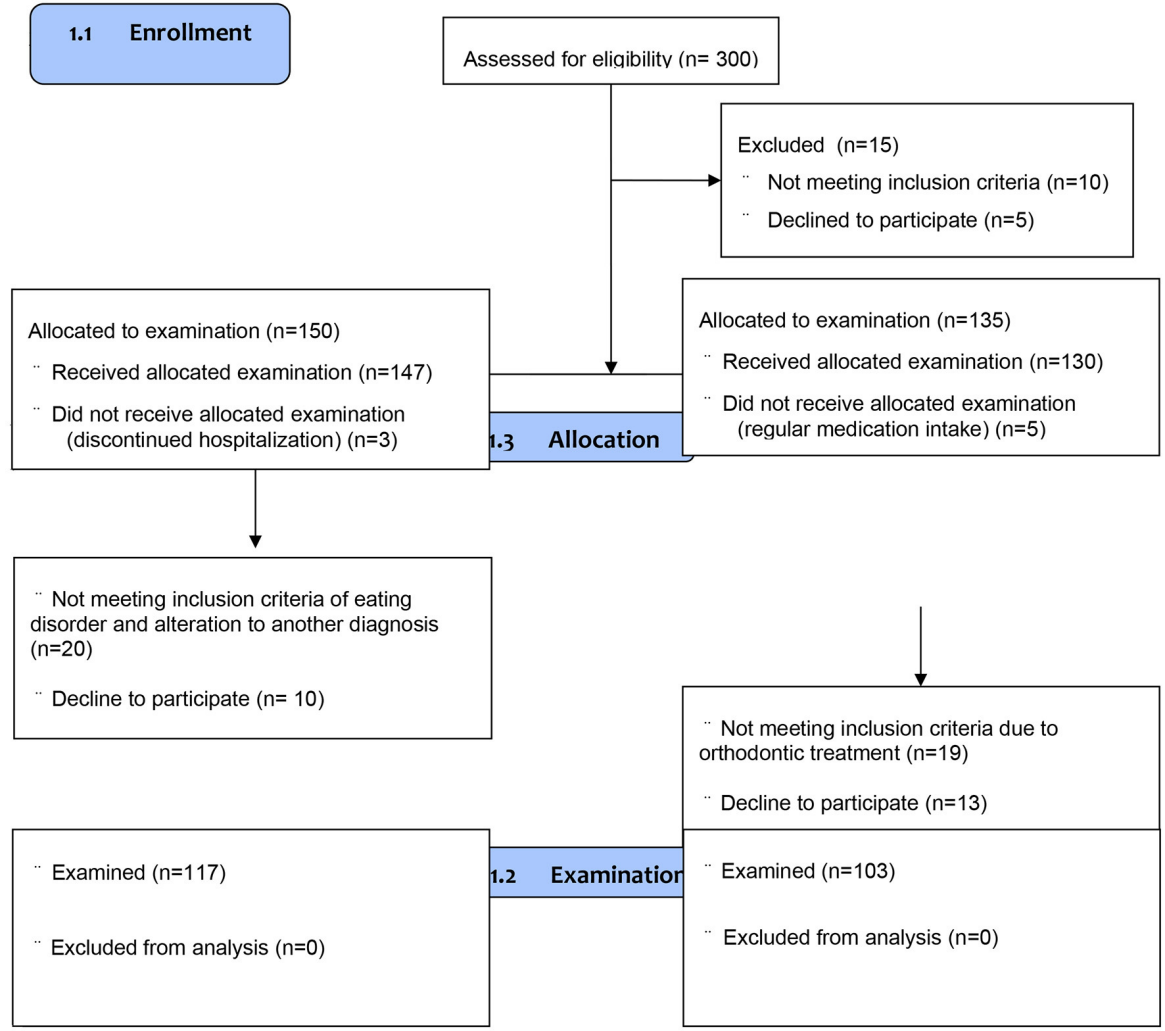
Flow chart of the study.

The control group (Ctrl group) consisted of one-hundred-three female teenagers recruited among one-hundred-thirty dental patients attending routine dental care in the public University dental clinic matched in terms of age (12–18 years) and sex (female) in respect to the studied AN group in the same period. The control group consisted of eumenorrheic and generally healthy girls who agreed to participate in the study. Patients attending for urgent or non-routine treatment were excluded from the control group. Notably, no control subject reported any eating disorders in the past. Other exclusion criteria in the Ctrl group related to general health were: chronic somatic diseases, mental or neurodevelopmental disorders, hereditary disorders (first-degree relatives), pregnancy or breastfeeding, pharmacotherapy, hormonotherapy, dietary supplements, or contraception (Table 1). We found the following potential confounders: social status, uncontrolled diet, number of meals, mineral composition of teeth, the buffer capacity of saliva, oral pH, the frequency of tooth brushing, and using different oral hygiene products. Additionally, eligible volunteers were excluded if they were children of dental professionals/dental students and had received any general or oral care that could bias their gingival status (Table 1). The flow chart of the study is described in Figure 1.

Anthropometric measurements (height, weight) and an assessment of oral condition were taken within the patients' 1st week after admission of malnourished AN patients. Body height was measured with a SECA 216 wall stadiometer with an accuracy of 0.1 cm while the child was standing (14, 15). Bodyweight was recorded in light clothing on a digital scale with an accuracy of 0.1 kg (14, 15). Body mass index (BMI) as ratio of body weight (kg) to (height)^2^ and percentage of ideal body weight (% IBW) as ratio of actual to ideal weight (IBW) × 100% where IBW (kg) = height (cm) – 100 – {[(height (cm) – 150)]/2} according to the Lorentz formula (14–16).

### Clinical Dental Examination

The clinical dental examination included such elements as oral hygiene and gingival status regarding inflammation, evaluation of dental tissues using standardized indicators of dental caries, and erosive tooth wear (see description below). Before the study, two qualified dentists were trained and calibrated to a gold standard (EP, AH). Examiner reliability was acceptable for the oral examination parameters because the ICC values and Cohen's Kappa coefficient were ≥0.9 (*p* < 0.001).

Dental plaque and gingival conditions were recorded using a manual graded periodontal WHO probe (LM-instruments, LM8 5050 probe, Osakeyhtiö, Parainen, Finland). The probe consisted of a 0.5 mm ball at the tip and had mm markings at 3.5, 8.5, 11.5 mm and color-coding from 3.5 to 5.5 mm. The probing was performed using only gentle probing forces with a periodontal probe of suitable dimensions (force 0.25–0.30 N). Plaque control was evaluated using the dichotomized Plaque Control Record index (PCR) (17). Gingival inflammation was determined using the Bleeding on Probing index (BOP) (18), measured in six points of the gingival sulcus of all teeth (excluding the third molars) by the same WHO probe. The proportion of surfaces (%) with dental plaque or bleeding-on-probing gums was calculated as % of sites (17–19).

After cleaning and drying (excluding the third molars), the teeth surfaces were scored under good dental lighting, without magnification (20). Dental examination records included the number of carious teeth, the number of restored teeth by fillings, and the number of missed teeth due to caries, using the Decayed, Missing, Filled teeth (DMFT) score evaluating dental caries (20).

The dental examination also included erosive tooth wear by Basic Erosive Wear Examination (BEWE) score (21). The BEWE is a partial scoring system that recorded the most acutely affected accessible dental surfaces (buccal, lingual, occlusal) in a sextant. Criteria for erosive wear estimation were in the following grades: 0—erosive tooth wear absent; 1—initial loss of dental structure (dentine not involved); 2—significant hard tissue loss <50% of the surface area (dentine involved); 3—significant hard tissue loss higher than 50% of the surface area (dentine involved) (22).

### Statistical Analysis

As appropriate, the analyzed data were expressed as mean ± standard deviation, median, minimum and maximum values, interquartile range, or percentage. Normality of distribution was tested using the Shapiro–Wilk test (interval scale). Two unpaired groups were compared using the Mann–Whitney U-test (data were not normally distributed or ordinal data). The relationship between variables was analyzed with Spearman's rank correlation coefficient (when data were not normally distributed or ordinal data). Categorical data were analyzed with the χ^2^ test or the Fisher-Freeman-Halton test (for contingency table larger than 2 × 2 with any expected values was less or equal to 5). Statistical analyses were performed with STATISTICA 13.0 (StatSoft Inc., Tulsa, USA) or StatXact 11.0 (Cytel Inc., Waltham, Massachusetts, USA). Multivariate analysis as logistic regression (backward, forward) was also carried out to determine the AN group's risk factors. The odds ratio and 95% confidence intervals were set for the indicated variables. This way, the answer to the question was which of the independent variables significantly influences the AN group. Therefore, a relationship was sought between the probability of disease occurrence and the group of independent variables. The parameters taken for analysis were selected following previous research and the observations found in the literature. The variables included in the logistic regression were: BMI, duration of AN illness, DMFT, PCR, BOP, and BEWE. Logistic regression calculations and intra-examiner calibration results (ICC) and Cohen's Kappa coefficient were performed in a statistical package MedCalc v. 19.5.1 (MedCalc Software, Ostend, Belgium). All results were considered significant at *p* < 0.05.

Considering the size of the target AN population, the sample size was based on European data between 8 and 13 AN cases on 100,000 adolescent females. Similarly, in Poland, AN's frequency is estimated between 0.8 and 1.8% of girls under 18 y.o. (23, 24). It was calculated using Cochran's formula (25) that at least 36–59% of the target AN eligible population individuals should be surveyed to reach a margin level of 2% at the confidence level of 95%.

The reporting of the study was made according to the Strengthening the Reporting of the Observational Studies in Epidemiology (STROBE) guidelines (Supplementary Material).

## Results

### Demographic and Clinical Characteristics

All details of socio-demographic data, height, weight, BMI, and IBW% are presented in Table 2A. The mean age of all participants was 15.1 ± 1.8. No significant differences were found in age between all groups (p = 0.746). BMI was in the normal weight range for the Ctrl group (19.8 ± 2.3), while it was under the threshold of 15 kg/m^2^ (severe underweight) for the AN group (14.2 ± 1.5, *p* < 0.001). Participants in the AN group were 32% lighter in weight than those in the healthy Ctrl group (*p* < 0.001). AN individuals suffered from eating disorder symptoms for at least < 12 months; the mean duration time was 11.1 ± 7.2 months.

**Table 2A:** Comparison of anthropometric and oral parameters between AN patients and controls.

**Group variables**	**All *n* = 220**	**AN *n* = 117**	**Ctrl *n* = 103**	***p*-value**
Age	15.1 ± 1.8	14.9 ± 1.8	15.0 ± 1.8	0.746 (ns)
Weight (kg)	44.9 ± 11.1	37.0 ± 5.5	53.9 ± 8.7	<0.001
Height (cm)	162.5 ± 7.3	161.1 ± 7.6	164.1 ± 6.6	<0.001
BMI (kg/m^2^)	16.8 ± 3.6	14.2 ± 1.5	19.8 ± 2.3	<0.001
IBW (%)	65.6 ± 13.1	56.2 ± 7.5	76.1 ± 9.7	<0.001
Duration of AN disease	–	11.1 ± 7.2	–	–
Number of teeth	27.7 ± 1.3	27.6 ± 1.8	27.8 ± 0.5	0.837 (ns)
D	0.7 ± 2.0	1.2 ± 2.6	0.1 ± 0.4	<0.001
	**0** (0–21)	**0** (0–21)	**0** (0–2)	
M	0.1 ± 0.3	0.1 ± 0.5	0.0 ± 0.0	0.017
	**0** (0–3)	**0** (0–3)	**0** (0–1)	
F	2.2 ± 3.0	2.5 ± 3.6	1.8 ± 2.0	0.914 (ns)
	**1** (0–16)	**1** (0–16)	**1** (0–10)	
DMFT	2.9 ± 3.7	3.8 ± 4.5	1.9 ± 2.1	0.005
	**2** (0–21)	**2** (0–21)	**2** (0–10)	
PCR (% of sites)	29.7 ± 25.0	43.8 ± 23.4	13.7 ± 15.4	<0.001
	**25** (0–100)	**40** (10–100)	**10** (0–54)	
BOP (% of sites)	12.5 ± 17.6	20.0 ± 20.1	3.9 ± 8.1	<0.001
	**5** (0–90)	**15** (0–90)	**0** (0–35)	
BEWE *n* (%)	25 (21.7)	22 (18.9)	3 (2.9)	<0.001
≤ 2	4 (3.9)	1 (1.0)	3 (2.9)	
3–8	19 (16.2)	19 (16.2)	0 (0)	
9–13	2 (1.7)	2 (1.7)	0 (0)	
≥14	0 (0)	0 (0)	0 (0)	

*Description of the abbreviations: mean ± standard deviation SD; Median (Minimum–Maximum); ns – not significant; duration of AN disease is expressed in the number of months*.

Although cell blood tests still showed a satisfactory WBC level in AN groups, neutrophiles maintained under a lower reference level (Table 2B).

**Table 2B:** Socio-economic status, education, blood parameters among AN group.

**Group variables**	**AN** ***n*** **=** **117**		
Duration of school education (y.)	8.7 ± 1.6 **9** (6–11)	First menstruation (age)	12.12 ± 1.12 **12** (11–15)
Mother graduated education	**3** (1–4)	WBC	5.34 ± 1.76 **5.1** (2.4–11.1)
Father graduated education	**3** (1–4)	NEU	↓1.96 ± 1.14 **1.89** (0.57–5.36)

*Description of the abbreviations: mean ± standard deviation SD; Median (Minimum–Maximum); y.-years; parents graduated education division (1) Primary, (2) Vocational, (3) Secondary, (4) Higher; WBC- white blood cells were the reference level (reference values 4–10,000/ul; NEU-blood neutrophils level was decreased (reference values 2,500–5,000/ul)*.

In both groups (AN and controls), there was no presence of primary teeth. We performed an oral examination for gingival bleeding and dental parameters recording on permanent teeth. The most relevant findings of the present study were the tendencies for dental caries, and poor oral hygiene connected with gingival bleeding detected in the oral cavities of anorexic patients compared with those in the Ctrl group. Dental examination indicated that 37.6% of AN patients vs. 11.7% of the controls were affected by dental caries. Continuously there was a significantly higher DMFT score than in the controls (3.8 ± 4.5 vs. 1.9 ± 2.1, *p* < 0.005), as well as the number of decayed teeth (1.2 ± 2.6 vs. 0.1 ± 0.4, *p* < 0.001), the number of missing teeth (0.1 ± 0.5 vs. 0, *p* < 0.02) (Table 2A).

There was a significant difference in prevalence and severity of erosive tooth wear. A BEWE score ≤ 2 was detected in 18.9% of AN patients as compared with 2.9% Ctrl subjects (*p* < 0.001) but no present any higher score ≥ 3 (0% among Ctrl group) (Table 2A).

Regarding periodontal parameters, the mean percentages of sites with dental plaque and bleeding on probing were significantly higher among AN patients than in controls (43.8 ± 23.4 and 20.0 ± 20.1 vs. 13.7 ± 15.4 and 3.9 ± 8.1, *p* < 0.001, twice respectively) (Table 2A).

Comparison of PCR, BOP and BEWE in AN subgroups divided according to vomiting incidents showed significant differences in worse results for AN subgroups with purging episodes (*p* < 0.05; *p* < 0.005; *p* < 0.001, respectively). However, AN patients from the purging subgroup presented a higher BMI index and more extended AN disease history (15.0 ± 0.9, *p* < 0.05; 15.1 ± 8.1, *p* < 0.004, respectively) (Table 2C).

**Table 2C:** Comparison of duration of disease, BMI, PCR%, BOP%, BEWE in AN subgroups according to purging episodes.

**Subgroup variable**	**AN without purging episodes *n* = 99**	**AN with purging episodes *n* = 18**	***p*-value**
BMI	14.1 ± 1.5	15.0 ± 0.9	<0.05
	**14.2** (10.7–19.1)	**14.8** (13.7–16.6)	
Time duration of AN disease (months)	10.1 ± 5.9	15.1 ± 8.1	<0.004
	**8** (2–36)	**12** (6–36)	
PCR (% of sites)	40.0 ± 22.2	59.8 ± 32.4	<0.05
	**37.5** (10–90)	**50** (15–100)	
BOP (% of sites)	15.3 ± 18.0	41.1 ± 30.6	<0.005
	**10** (0–60)	**35** (0–90)	
BEWE	0.3 ± 1.3	5.6 ± 2.5	<0.001
	**0** (0–8)	**5** (3–12)	

*Description of the abbreviations: mean ± standard deviation SD; Median (Minimum–Maximum); ns – not significant; duration of AN disease is expressed in the number of months*.

### Correlations Between Analyzed Variables

In the AN group, Spearman analysis showed correlation between anthropometric measurements as percentage of ideal body weight (% IBW) and blood tests as WBC (r_s_ = 0.39; p = 0.009), neutrophile level (r_s_ = 0.38; p = 0.011). As to neutrophile level there was correlation found to PCR (r_s_ = 0.32; p = 0.031) and BOP (r_s_ = 0.30; p = 0.047). Other associations evidenced a significant relationship between duration of AN disease and BOP (r_s_ = 0.19; p = 0.036), BEWE (r_s_ = 0.28; p = 0.002). Moreover, PCR was correlated to the number of decayed teeth (r_s_ = 0.41; p = 0.0001), the number of restorations F (r_s_ = 0.29; p = 0.002), DMFT (r_s_ = 0.49; p = 0.0001) and BOP showed significant association with the number of decayed teeth D (r_s_ = 0.42; p = 0.0001), number of restorations F (r_s_ = 0.29; p = 0.001), DMFT (r_s_ = 0.49; p = 0.0001), PCR (r_s_ = 0.92; p = 0.0001). The correlation was also observed in tooth wear score BEWE and PCR (r_s_ = 0.21; p = 0.033) and BOP (r_s_ = 0.24; p = 0.010).

In the AN group, regarding the socio-economic relationship Spearman analysis showed a significant association between mother's education level and DMFT index (r_s_ = 0.39; p = 0.022). Father's education level was correlated to the number of missing teeth M (r_s_ = −0.38; p = 0.027), PCR (r_s_ = 0.35; p = 0.041) and BOP (r_s_ = 0.39; p = 0.024).

**Figure 2:**
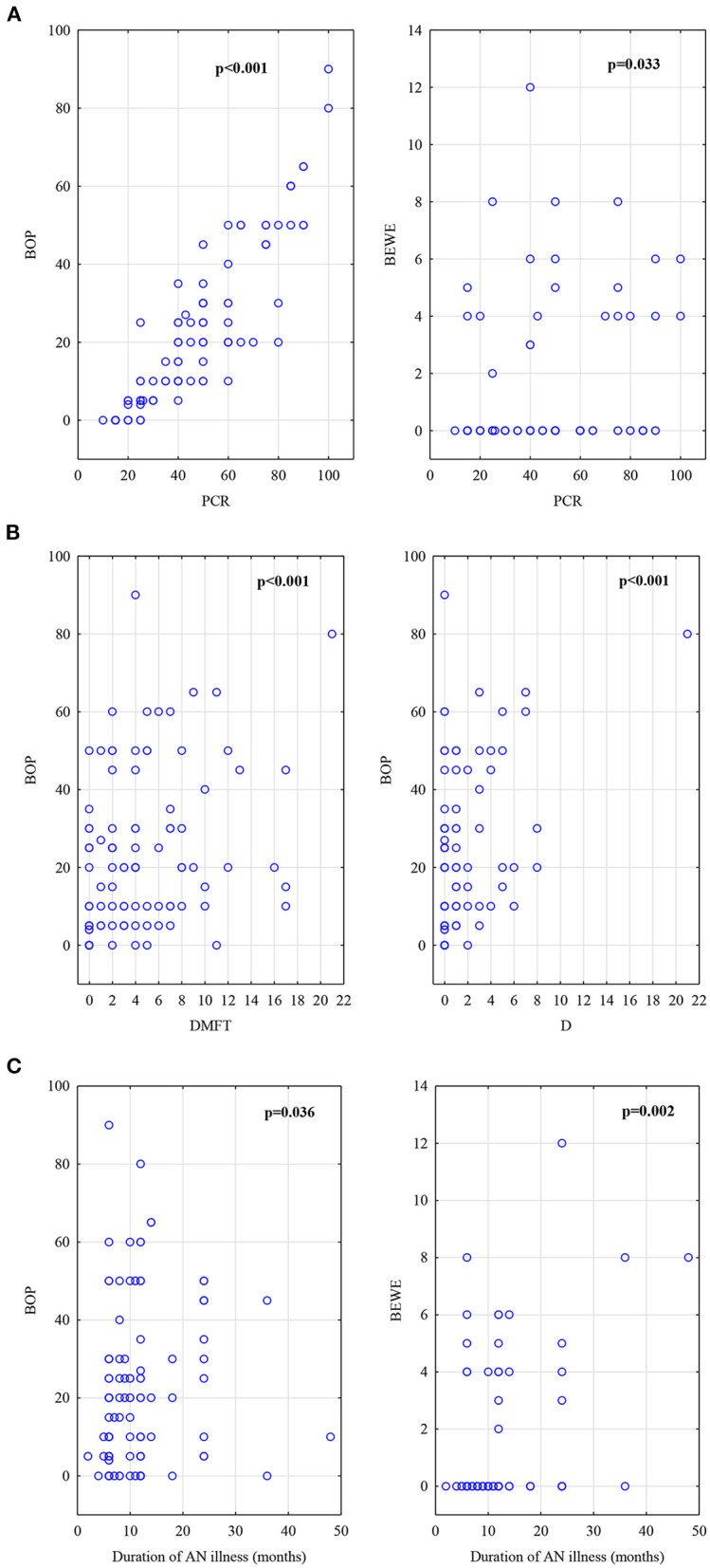
**(A)**Correlations in AN group, BOP index and BEWE index to PCR index. **(B)** Correlations in AN group, DMFT score and D number to BOP index. **(C)** Correlations in AN group, BOP index and BEWE index to duration of AN illness (counted in months).

The AN group's main correlations are shown in Figures 2A–C.

In the Ctrl group statistical analysis showed significant correlations between anthropometric and oral parameters such as age and DMFT (r_s_ = 0.37; p = 0.0002) and BEWE score (r_s_ = 0.24; p = 0.016). The BMI was correlated to the number of decayed teeth (r_s_ = 0.21; p = 0.042), filled teeth (r_s_ = 0.32; p = 0.001), DMFT (r_s_ = 0.35; p = 0.0003), BOP (r_s_ = 0.21; p = 0.037). There was also a relationship between the number of carious teeth and dental plaque as PCR (r_s_ = 0.25; p = 0.012), BOP (r_s_ = 0.33; p = 0.014). Dental plaque as PCR% and BOP% revealed a distinct association (r_s_ = 0.67; p = 0.0001).

From the group of variables included in the logistic regression: BMI, duration of AN illness, DMFT, PCR, BOP, BEWE, the multivariate analysis model indicated three variables: BMI, DMFT, PCR, as they turned out to be statistically significant at the level of *p* < 0.05. The odds ratio (OR) of the BMI parameter was 0.07, which means that as the BMI increases, the chance of getting ill (being in a group of AN) decreases. For DMFT, PCR the odds ratio was 1.81 and 1.08 (OR > 1), i.e., an increase by a DMFT index, PCR causes almost two times and 1.1 times higher a chance of being in the AN group, compared to the control subjects (Tables 3A,B).

**Tables 3A and B:** The logistic regression results indicated three variables: BMI, DMFT, and PCR. They turned out to be statistically significant at *p* < 0.05.

**(A) Coefficients and standard errors**				
**Variable**	**Coefficient**	**Std. error**	**Wald**	**p**
PCR	0.08	0.03	7.02	0.008
DMFT	0.59	0.29	4.21	0.041
BMI	−2.62	0.67	15.51	0.0001
Constant	40.21	10.52	14.61	0.0001
**Variables not included in the model**				
BEWE				
BOP%				
**(B) Odds ratios and 95% confidence intervals**.				
**Variable**	**Odds ratio**	**95% CI**		
PCR	1.08	1.02 to 1.15		
DMFT	1.81	1.03 to 3.15		
BMI	0.07	0.02 to 0.27		

*PCR, Plaque Index Record; DMFT-Decay, Missing and Filling Teeth index; BMI, Body Mass Index; BOP%, Bleeding on Probe index*.

## Discussion

In our study, patients from the AN group presented a higher incidence of oral-related complications regarding dental status and erosive tooth wear, less efficient control of plaque, and gingival inflammation than healthy subjects. A novel of our report is a collection of AN subjects in the youngest age under 18-years-old. After reviewing the literature on EDs, it should be noted that most oral clinical trials involved adult female patients (over 20 years of age) with a longer duration of AN than 5 years. Eating disorders are thus chronic diseases and are often associated with taking antidepressants and psychotropic drugs (26). Few dental analyses were based on a younger population of patients; the majority were limited in the number of cases and included a wide age range from 18 to 60-years-old (9, 27–40). Based on the analysis of the scientific literature, we found only nine studies separately involving young AN patients aged 18-years-old, as shown in Table 4 (8, 27–34). The rest of the published studies related to the symptoms in the oral cavity of various types of EDs or AN patients represented <10% study group (9, 35–40). Selected results from 9 original studies indicated a significantly worse dental status in AN patients, which increased with their age and disease duration (8, 27–34). This is also in line with the literature reviews on the ED and oral implications (9, 41, 42).

**Table 4:** A synthesis of data obtained from the electronic research organized in PubMed database and Web of Science.

**Clinical study (publishing year)**	**Number of AN patients (subtype)**	**Age range min-max or mean ± SD (years)**	**Control group +yes –no**	**Oral examination**	**Significant results**
Hellström (27)	12 (restricting) 27 (purging)	rr 14–42 24.5 ± 1.3 26.2 ± 1.2	–	Caries, plaque, gingivitis, erosion, saliva analyses (secretion,pH, buffering)	More frequent erosion in purging type
Touyz et al. (28)	15	20.1 ± 8.3	+	DMFT, plaque, CPITN, erosion, recessions, salivary secretion, pH	Less plaque (51%) but more frequent gingivitis (16.9%) and recessions (10.2%), lower saliva pH (7.1 ± 0.4)
Daszkowska et al. (29)	15 (restricting) 35 (purging)	11.3–21.6	+	DMFT, caries frequency, erosion	94.3% caries frequency in restricting subgroup, vomiting was associated with greater erosion
Shaughnessy et al. (30)	23	rr 14.4–7.2	–	DMFT, OHI-S, erosion, recessions, MCW	DMFT (8.6), OHI-S: very good to excellent oral hygiene, recessions in 43% of AN subjects, no erosion detected (ns), MCW (4.8 mm)
Back-Brito et al. (31)	11 (restricting) 21 (purging)	rr 19–58 mean 26	+	Fungal flora	Common results calculated together with 27 BN subjects observed greater percentage for Candida species (74.6%)
Johansson et al. (32)	14	rr 10–50	+	DMFT, VPI, GBI, erosion, salivary secretion, soft tissue lesions	Common results calculated together with 8 BN and 32 EDNOS subjects as eating disorders (ED): lower VPI (7.1%) and GBI (1%), higher erosion, incidence of soft tissue lesions
Lourenço et al. (33)	18	rr 18–50	+	DMFT, gingivitis, periodontitis, erosion, salivary secretion, soft tissue lesions	Common results calculated together with 15 BN as ED: higher DMFT (8.8 ± 7.0), periodontitis, erosion, self-reported dentin hypersensitivity, incidence of soft tissue lesions, lower salivary flow rate
Garrido-Martínez et al. (34)	1 (restricting) 15 (purging)	rr 19–44 mean 27.6	+	DMFT, PI, erosion, salivary secretion, soft tissue lesions	Common results calculated together with other 43 ED patients: lower salivary flow rate, higher erosion and incidence of soft tissue lesions
Pallier et al. (8)	36	32.1 ± 9.1	+	DMFT, PI, BOP, BEWE.	Higher PI (78.8 ± 19.7%), BOP (41.3 ± 27.2%) and BEWE > 3 (41.7%)

*Description of the abbreviations: mean ± standard deviation SD; rr, range of age as min-max; ns, not significant; BEWE, Basic Erosive Wear Examination; BN, bulimia nervosa; BOP, Bleeding on Probing; CPITN, Community Periodontal Index of Treatment Needs; DMFT, Decayed, Missing, and Filled Teeth; EDNOS, eating disorder not otherwise specified; GBI, Gingival Bleeding Index; GI, Gingival Index; MCW, mandibular cortical width; OHI-S, Simplified Oral Hygiene Index; PI, Plaque Index; VPI, Visual Plaque Index*.

*The following MeSH and non-MeSH search terms were used: (“Feeding” [MeSH terms] OR “Eating Disorders” [All fields] OR “Oral Health” [All fields]). “Feeding” and “Eating disorders” include the following terms: Anorexia Nervosa, Avoidant Restrictive Food Intake Disorder, Binge-Eating Disorder, Bulimia Nervosa, Diabulimia, Feeding and Eating Disorders of Childhood, Food Addiction, Night Eating Syndrome, Pica, Relative Energy Deficiency in Sport, Female Athlete Triad Syndrome, Rumination Syndrome. The search selected publications only with an anorectic group of patients published in English (9, 27–34)*.

Our subjects from the study group were young female AN patients up to 18 years of age in whom the disease only first developed. A dentist's support could counteract side effects and thus prevent permanent changes in oral homeostasis, which is also crucial for their health in the future (43–51). In the case of ED, including AN, the disease symptoms at an early age give an uncertain prognosis and reduce the chances of success in psychiatric therapy (3). The same thesis seems to apply to oral health. While in the case of the non-physiological destruction of teeth such as dental attrition or abrasion, implementation of changes in the patient's dental habits may stop tooth wearing. Unfortunately, the protection of teeth against caries or erosion is more multivariate, as it is often not conducive to the patient's general condition (44–52), selective low pH diet (52, 53), the insufficient activity of the salivary glands (6–8), salivary buffering capacity (54). Moreover, the dentist's commitment to dental patients affected by AN may motivate them to undertake general treatment and combat the disease (41, 42). Multidisciplinary support in the case of patients' severe psychosomatic conditions seems to be not only an empathetic or ethical but definitely a therapeutic indication (55, 56). Nevertheless, the multidisciplinary assessment of the AN adolescent population in longitudinal studies may be suggested for future research.

The mean DMFT score among AN subjects was significantly higher than the control group in the presented study. Other studies conducted on adult AN patients are similar (8, 33, 48). However, DMFT values usually reported higher numbers due to the older age of enrolled patients (>20-years-old), the chronic character of the disease (>5 years earlier AN diagnosis before oral examination), and psychotropic pharmacotherapy, which may limit salivary flow and make easier dental plaque colonization (8, 32, 48). Compared to a nationwide epidemiological oral health survey conducted in Poland among healthy age groups (15-year-olds, *n* = 2,000), the DMFT index among girls was estimated at the same level or higher than the obtained results (57–59). However, it may be commented that additional factors could influence the DMFT results as the different countryside living locations, mineral composition, the buffer capacity of saliva secretion, the frequency of tooth brushing, and the number of consumed meals, including carbohydrates (57–59).

The assessment of the BEWE index showed a low risk of dental erosion. It is worth noting that AN young patients (symptoms of ED had been observed for an average of 12 months) reported rare vomiting inducing. We suspect that the purging episodes had to occur much less frequently than in patients with bulimia nervosa (BN), in whom the severity of erosion is reported in the literature from 30 to 70% (49, 50, 52, 60–62). Pallier et al. (8) found a high risk of erosion in 16.7% of AN patients, but they studied much older adult female patients with long-term, chronic disease. However, the AN subgroups with purging episodes showed worse results concerning dental erosion. Apart from vomiting, more factors influence the BEWE score. The role of an acid diet may be crucial; foods and drinks predispose to decrease the pH of the oral cavity, especially in persons with severe ED when unstimulated saliva secretion is diminished (52, 53, 60, 61). Finally, it may also be connected with loss of hard tissues due to accompanying attrition or abrasion (62, 63). Shellis and Addy (63) pointed out that these processes coexist and have strong relationships. Comparing the findings on erosive changes among healthy 15-year-olds from southern Poland (*n* = 181), Kaczmarek et al. (62) observed only initial enamel erosion and more often observed it in boys than in girls. It is suggested that a low pH diet, especially drinks, and excessive physical activity may limit salivary buffer capacity at this age and stimulate non-carious dental tissue loss (64, 65).

It should be noted that AN subjects often perform excessive physical activities and prefer acid beverages, but they limit the amount of food consumption (32). The abovementioned data showed that dental erosion might occur among adolescents in various degrees of tooth wear with age. It is vital to adopt the hypothesis about the potential incidence of erosion among children (65).

In the AN group, poor dental hygiene and gingival inflammation are in line with other ED reports (11, 28, 32, 37, 40). In comparing our research with a national survey among healthy adolescents aged 16–18 years, Pietrzak et al. reached better dental hygiene results (66). We may explain our worse results; those dental examinations carried out in the first week after admission to the hospital ward might impact the worse oral hygiene conditions. The beginning of the hospital treatment seems to be a challenging period for AN patients, as they are mainly weakened and, at the same time, subjected to additional stress related to the hospital stay. All inpatients were enrolled in a behaviorally oriented nutritional rehabilitation program. They ate meals under the supervision of nursing personnel for 1-h observation. Thus, they could not perform hygienic procedures immediately after eating. Due to the nature of the therapy, meals contained a lot of simple sugars with a sticky consistency (e.g., jams, sweetened tea, white bread). Based on our observation and literature data, poor motivation to maintain good oral hygiene may also be influenced by a difficult life situation and the related apathy, depressed mood, psychomotor drive, and suicidal tendencies (67, 68). Other investigations indicated that a diet low in protein, vitamins, and unsaturated fatty acids contributes to metabolic and biochemical disturbances that cause an imbalance between oxidants and antioxidants (5–7, 53, 69). We agree with other authors that the pervasive state of malnutrition, especially in highly severe AN, influenced medical complications and a risk of rapid gingival inflammation (8, 9, 34). Obtained correlations to PCR and BOP indexes with lower neutrophile count results may suggest a relationship to the risk of gingival inflammation. It is worth quoting from other laboratory studies that about 80% of AN patients are affected by anemia, leukopenia 29%, neutropenia 79%, thrombocytopenia 25%, and 17% develop thrombocytosis (70). In our study, severe malnutrition and the laboratory-tested neutrophiles may explain the relationship between nutritional deficiencies and body defense and susceptibility to gingival complications (71, 72).

**Figure 3:**
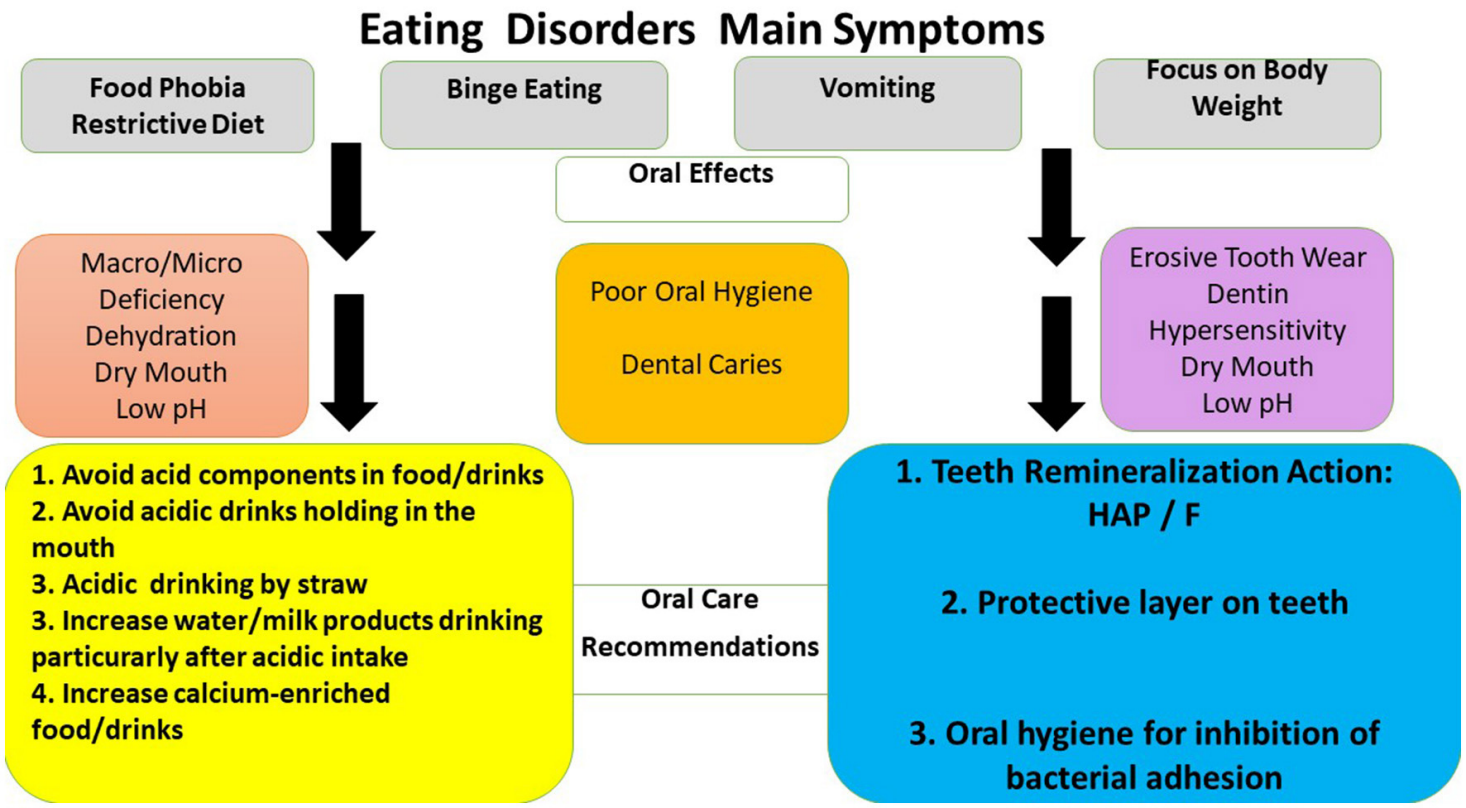
Oral effects of eating disorders and oral care recommendations to health professionals and patients. HAP- oral care products containing synthetic hydroxyapatites, CPP-ACP- oral care products containing calcium-phosphate agents, F - oral care products containing fluoride products (73, 74, 76, 79).

Consensus Report of the European Federation of Conservative Dentistry on eating disorders concluded that dental caries and erosion prevention objectives reduce or arrest the progression of these two processes, screening and monitoring recall dental sessions (73). Our dental data indicated that the main tasks should include three directions: oral hygiene, remineralization, and diet advice (73–78). Figure 3 shows that all preventive approaches may be applied (73, 79).

The present study's authors are aware of its limitations and recall most of them. The participants were not checked whether gastroesophageal reflux (GERD) or any other condition could have exposed their teeth to intrinsic sources of acid other than purging behavior. However, a qualified physician excluded any somatic diseases during a diagnostic interview. All patients were asked to express any digestive system ailments and did not report any symptoms. It is worth noting that all patients were hospitalized the first time after the onset of the AN disease. At this phase, a crucial hospitalization role was making the correct diagnosis but detecting any additional symptoms. The final diagnosis of all patients was the restrictive subtype of AN.

In addition, we can assume that the subjects had no comparable diet habits due to AN disease. It would be worth analyzing nutritional intake according to quantity and quality important from the point of view of oral health safety. According to diet behaviors in early disease development, anorexics do not have the same opinion on diet preferences. They often claim that they eat larger meals than before and start a selective diet. However, it is not easy to rely on such data. Studies have shown that their diet is low in carbohydrates (eliminated as the first ingredient). At the same time, protein and fat intake are maintained at an acceptable level, especially at the beginning of the disease (71, 80, 81). Besides protein-energy malnutrition (PEM), any deficiencies related to counts may raise the risk of bacterial infection.

It seems essential to consider a significant risk factor for erosive tooth wear among adolescents with purging/bulimic symptoms. Professional food and drink advice may have a preventive role to avoid erosive tooth wear (79). A significant limitation was a lack of data according to earlier oral status in both AN and Ctrl groups. This comparison could give additional conclusions for all children suffering from ED. Longer follow-up periods may be suggested to evaluate the clinical approach to oral health among AN patients.

Nevertheless, one may argue that we examined a small cohort of young AN patients under real-life conditions. Besides, family members were not included in the dental examination because living a long distance from the hospital clinic. Moreover, our project was also refined by the case-control design, and the assessment of oral clinical markers was based on visual criteria when examining the oral cavity. In detecting and assessing caries, digital devices could be implemented, e.g., DIAGNOdent Pen, DIAGNOcam and CarieScan PRO, but the visual DMFT index makes the results comparable to previously performed studies that focused on ED and AN. To our knowledge, this is the first clinical trial that compared such a group of AN patients in terms of homogeneity of subtype symptoms, adolescent age, sex, living area, nutritional status, and duration of disease, fewer than 5 years.

Even if our results are not enough to prove oral conditions among anorexic subjects, our limitations do not undermine the representativeness of the examined group.

## Conclusions

Determination of AN restrictive subtype in adolescence may indicate numerous oral-related complications from dental caries, the beginning of erosive tooth wear, gingival inflammation development, and failure to cope with dental hygiene. Although the obtained results did not reveal any severe oral condition, there is a need for adequate oral hygiene/diet instructions combined with regular oral care to prevent forward complications in the patients' future.

Dental problems need to be tackled together with medical insight and interaction with physicians in the future. These disturbances to oral health should be routinely investigated, monitored, and treated during the perception of the primary disease. Multidisciplinary management of AN is essential to prevent the chronicity of the disorder and the potential severity of negligence.

## Data Availability Statement

The raw data supporting the conclusions of this article will be made available by the authors, without undue reservation.

## Ethics Statement

The studies involving human participants were reviewed and approved by Bioethics Committee of the Poznan University of Medical Sciences (Resolution No. 66/12). Written informed consent to participate in this study was provided by the participants' legal guardian/next of kin.

## Author Contributions

EP, MT-N, and AS contributed to the conception and design of the study. AH, MR, KJ, and EP organized the database. MR performed the statistical analysis. EP performed clinical examinations. All authors contributed to manuscript revision and read and approved the submitted version.

## Funding

Poznan University of Medical Sciences handed over funding sources for the research.

## Conflict of Interest

The authors declare that the research was conducted in the absence of any commercial or financial relationships that could be construed as a potential conflict of interest.

## Publisher's Note

All claims expressed in this article are solely those of the authors and do not necessarily represent those of their affiliated organizations, or those of the publisher, the editors and the reviewers. Any product that may be evaluated in this article, or claim that may be made by its manufacturer, is not guaranteed or endorsed by the publisher.
