# Corrigendum: Risk of Dental Caries and Erosive Tooth Wear in 117 Children and Adolescents' Anorexia Nervosa Population—A Case-Control Study

**DOI:** 10.3389/fpsyt.2022.956739

**Published:** 2022-07-08

**Authors:** Elzbieta Paszynska, Amadeusz Hernik, Agnieszka Slopien, Magdalena Roszak, Katarzyna Jowik, Monika Dmitrzak-Weglarz, Marta Tyszkiewicz-Nwafor

**Affiliations:** ^1^Department of Integrated Dentistry, Poznan University of Medical Sciences, Poznań, Poland; ^2^Department of Child and Adolescent Psychiatry, Poznan University of Medical Sciences, Poznań, Poland; ^3^Department of Computer Sciences and Statistics, Poznan University of Medical Sciences, Poznań, Poland; ^4^Department of Psychiatric Genetics, Department of Psychiatry, Poznan University of Medical Sciences, Poznań, Poland

**Keywords:** anorexia nervosa, adolescence, oral health, caries, erosion, dental plaque, gingival inflammation

In the original article, there was an error in the legend for Table 1 as published. The correct legend appears below. “AN, anorexia nervosa; ED, eating disorders; ICD-10, International Statistical Classification of Diseases and Related Health Problems (10th edition); DSM-V, Diagnostic and statistical manual of mental disorders (5th ed.); CNS, Central Nervous System; BMI, Body Mass Index.”

In the original article, there was a mistake in Table 1 as published. A correction has been made in the middle column entitled “Criteria for inclusion into the control group (Ctrl)”; the MINI-Kid questionnaire was not used for the criteria for inclusion into the control Group. The corrected Table 1 appears below.

**Table 1:** Inclusion and exclusion criteria for both groups.

**Criteria for inclusion into the study group (AN)**	**Criteria for inclusion into the control group (Ctrl)**	**Criteria for exclusion from study and control groups**
Children of female sex aged 12–18	Children of female sex aged 12–18	Adolescents aged > 18 Male sex
Children with diagnosed AN restrictive subtype in accordance with ICD-10 and DSM-V diagnostic criteria (diagnosis confirmed by two independent psychiatrists)	Lack of mental disorders—assessment with the use of ICD-10 and DSM-V diagnostic criteria (confirmed by two independent psychiatrists). Children without hereditary mental disorders (first-degree relatives)	Children with disorders of the central nervous system (e.g., epilepsy, serious injuries, and CNS infections) Coexisting: schizophrenia, bipolar affective disorder, any serious somatic disorders
Clinically significant AN symptoms lasting over six months	No ED symptoms in present and past times	Chronic somatic diseases Persistent pharmacotherapy Hormonotherapy Contraception Pregnancy Dietary supplements
BMI < 15 kg/m^2^	BMI 17–24 kg/m^2^	BMI > 25 kg/m^2^
A patient, parent or legal guardian approval	A patient, parent or legal guardian approval	Lack of acceptance from patients, parents or legal guardians
N smokers	No smokers	Smoking
	No urgent dental recall visit	Professional scaling Orthodontic treatment Antibiotic therapy Anti-inflammatory drugs 3 months before dental examination

*AN, anorexia nervosa; ED, eating disorders; ICD-10, International Statistical Classification of Diseases and Related Health Problems (10th edition); DSM-V, Diagnostic and statistical manual of mental disorders (5th ed.); CNS, Central Nervous System; BMI, Body Mass Index*.

In the original article, the reference for “Sheehan DV, Lecrubier Y, Sheehan KH, Amorim P, Janavs J, Weiller E, et al. The Mini-International Neuropsychiatric Interview (MINI): the development and validation of a structured diagnostic psychiatric interview for DSM-IV and ICD-10. *J Clin Psychiatry*. (1998) 59:22–33” was incorrectly written as reference 14. Reference 14 has been removed, therefore the numbering of the subsequent references is impacted. The original reference 15 is now 14, 16 is 15, 17 is 16, etc.

In the original article, there was an error in referencing. A correction has been made to citations in **Methodology**, *Participants*, paragraph two:

“…The AN group consisted of one-hundred-seventeen consecutive adolescent patients from the mid-west part of the country admitted in the acute phase of AN to one public Psychiatric Unit for Child and Adolescents from 2015 to 2020. Patients admitted to the hospital were primarily female because, during the survey, only one boy was hospitalized and suspected to be affected with AN. Still, he was excluded from the study group due to statistical reasons. Diagnosis of the restrictive subtype of AN was confirmed after a semistructured interview conducted by a child and adolescent psychiatrist based on ICD-10 (code F50.1) and DSM-5 (code 307.1) criteria (12, 13)…”

The authors apologize for these errors and state that they do not change the scientific conclusions of the article in any way. The original article has been updated.

## Publisher's Note

All claims expressed in this article are solely those of the authors and do not necessarily represent those of their affiliated organizations, or those of the publisher, the editors and the reviewers. Any product that may be evaluated in this article, or claim that may be made by its manufacturer, is not guaranteed or endorsed by the publisher.

